# How studies on zoonotic risks in wildlife implement the one health approach – A systematic review

**DOI:** 10.1016/j.onehlt.2024.100929

**Published:** 2024-11-08

**Authors:** Caroline Kuhn, Kenneth Mawuta Hayibor, Ama Twumwaa Acheampong, Luciana Salini Abrahão Pires, Magda Clara Vieira Costa-Ribeiro, María Soledad Burrone, Carlos Roberto Vásquez-Almazán, Katja Radon, María Teresa Solis Soto, on behalf of the KAPwildlife-study group, Abrahão Pires Luciana Salini, Adler Marcia, Burrone María Soledad, da Costa Ribeiro Magda Clara Vieira, de Almeida Gustavo Araújo, de Carvalho Denise Siqueira, de Tarso Pires Paulo, Encina Zamorra Veronica, Garrido Marie Astrid, Guzmán-Quilo Maria Carolina, Kuhn Caroline, Magalhães Buffon Marilene da Cruz, Mansilla Vivar Pilar Macarena, Mendez Heredia Dennis Martin, Perez Morales Fabiana Marcela, Pinto Navia Carlos Fernando, Radon Katja, Ribeiro de Almeida Tatjana Queiroz, Solis Soto María Teresa, Vásquez-Almazán Carlos Roberto

**Affiliations:** a Center for International Health at Ludwig-Maximilian-University Hospital, Munich, Germany; b Federal University of Paraná, Curitiba, Brazil; c Institute of Health Sciences, Universidad de O'Higgins, Rancagua, Chile; d Museo de Historia Natural, Escuela de Biología, Universidad San Carlos de Guatemala, Guatemala City, Guatemala; e CIHLMU OH TARGET Competence Center, Universidad San Francisco Xavier de Chuquisaca, Sucre, Bolivia

**Keywords:** One health, Zoonoses, Wild animals, Interdisciplinary research

## Abstract

**Background:**

The recent COVID-19 pandemic and the emergence of infectious diseases at the human-animal interface highlight the global challenge of mitigating zoonotic risks. The One Health approach emphasizes the interconnectedness of human, animal, and environmental health, urging for holistic and interdisciplinary strategies in disease prevention. Despite growing interest, the attention to wildlife in pandemic prevention remains limited. This systematic literature review aims to evaluate recent One Health research on zoonotic diseases and wildlife in terms of study design, interdisciplinary collaboration, and participatory approaches. Key questions addressed include the consideration of One Health domains, disciplinary involvement, and the inclusion of non-academic stakeholders.

**Methods:**

Following PRISMA guidelines, PubMed and Web of Science were searched for primary research papers on zoonotic diseases and wildlife from 2018 to 2023. Eligibility criteria included a focus on wildlife, zoonotic diseases, and adoption of the One Health approach.

**Results:**

A total of 228 primary research papers were retrieved. Out of these, 105 studies were included in the review. Few studies integrated human, animal, and environmental domains simultaneously in data collection (4.8 %) and knowledge generation (29.5 %). While extensive knowledge was generated for animal health (97.1 %) and human health (84.8 %), environmental health (34.3 %) remained underrepresented. Laboratory methods predominated (82.9 %), with limited integration of social science methodologies (19 %). The majority were epidemiological studies (86.7 %), yet analytical design within these was sparse (17.1 %). Participation of non-academic stakeholders was limited (36.2 % included non-academics; 3.8 % encompassed participative approaches).

**Conclusions:**

The synthesis of the domains human, animal and environmental health remained fragmentary in the studies reviewed. Environmental health is underrepresented and the interdisciplinary involvement of social sciences lacks. Neglecting these fields of competence impedes comprehensive understanding of disease dynamics and hampers effective zoonosis prevention strategies. In result, greater inter- and transdisciplinary collaboration, along with participatory approaches, are still needed for advancing One Health research.

## 1 Introduction

The emergence and transmission of zoonotic diseases pose a critical global challenge. Typically, wildlife populations act as reservoirs for zoonotic pathogens [1], while 72 % of zoonotic emerging infectious disease (EID) events originate from wildlife [2]. Recently, the One Health High-Level Expert Panel (OHHLEP) has highlighted the urgent need for action and prevention strategies, emphasizing the need for a holistic and interdisciplinary approach [3], and recommendations for zoonotic disease prevention measures draw attention to the One Health approach [4].

One Health is a holistic approach that addresses the interrelationships between human, animal, and environmental health. Underlying principles include inter- and transdisciplinarity. Interdisciplinarity involves two or more disciplines and focuses on integrating their perspectives, while transdisciplinarity goes beyond integration and is associated with practical solutions to a problem [5]. In this context, transdisciplinary research can be defined as research that goes beyond academia and involves non-academic stakeholders [6,7], including governmental and nongovernmental organizations and representatives of local communities [5]. Although the concept is not new, there is a growing interest in its implementation [8]. Particularly with regard to zoonotic diseases, One Health represents a promising shift to a comprehensive approach, as animal, human and environmental health cannot be considered separately. The added value of an integrated One Health approach compared to isolated approaches has been demonstrated in several studies, with particular emphasis on the integration of the One Health domains [9]. Qualitative benefits of the One Health approach include improved disease prediction, prevention and preparedness, inter- and transdisciplinary coordination, and advances in technology and diagnostics [9,10]. Quantitative benefits include reductions in disease incidence and burden [11] and potential economic benefits [[12], [13], [14]]. Further proof of concept and comparability of the One Health approach are needed [9,15,16]. Recently, great efforts have been made to bring human and animal health sciences and related institutions together at local, national, and international levels [17]. However, wildlife and the environment are still neglected in pandemic prevention [18]. The recent COVID-19 pandemic has highlighted the importance of human-wildlife interactions and the need for a more sustainable, less risky relationship with nature. Environmental change is discussed as the most critical cause of zoonotic EID [1]. Loss of biodiversity is a good example; areas under land use change bear the risk of pathogenic spillover and conversely, protecting biodiversity offers a high potential of zoonotic risk mitigation [19]. Furthermore, the environment plays an important direct or indirect role in transmission pathways. For example, cattle pastures and badger latrines have been identified as optimal environments for the transmission of mycobacteria between wildlife and livestock [20]. Environmental factors, such as climate change influence tick-borne diseases [21]. Especially in wildlife sciences an integrated approach, such as the One Health approach is considered critical [22]. In One Health research, the inclusion of correlations with other species or ecosystems improves the scientific quality [23]. Thus, it is essential to consider all One Health domains – human, animal and the environmental.

Reporting of data on all three domains and collaboration between different disciplines are key elements of One Health research, according to the Checklist for One Health Epidemiological Reporting of Evidence (COHERE statement) [24] and the framework proposed by Lebov et al. [25]. Furthermore, inter- and transdisciplinarity is essential to integrate knowledge and expertise from various fields and to foster innovation, synergy, and shared responsibility among stakeholders, ultimately promoting more sustainable and resilient health systems [10]. The Methodology for Interdisciplinary Research (MIR) framework can be consulted when planning and assessing collaborations, which suggests optimizing interdisciplinarity at three levels of research design: conceptual design, technical design, and integration [26]. In particular, we would like to highlight the inclusion of social sciences as a discipline with so far neglected potential within the One Health framework [27,28], and refer to the emphasis of decision-making and mutual ownership of the processes in participatory health research [29], which allows to strengthen the transdisciplinary character of One Health research.

There is a growing momentum to expand and enhance efforts in One Health research, underscored by initiatives such as the One Health Joint Plan of Action (OH JPA) of the Quadripartite Food and Agriculture Organization of the United Nations (FAO), United Nations Environmental Programme (UNEP), World Health Organization (WHO), and World Organization of Animal Health (WOAH) in 2022 [17]. With this systematic literature review, we aim to assess the status of One Health research on zoonotic diseases and wildlife in the recent years between 2018 and 2023 in terms of study design, holistic approach, interdisciplinarity, and transdisciplinarity. The review addresses the following questions:

1. In which One Health domains (human, animal, environment) were data collected or reported?
2. In which One Health domain was knowledge gained?
3. Which disciplines were involved in the study? Methodologies from which disciplines were adopted and brought together?
4. Did non-academics contribute to the study's results or interpretation? Is a participatory approach implemented in the study design?

## 2 Materials and methods

**Fig. 1:**
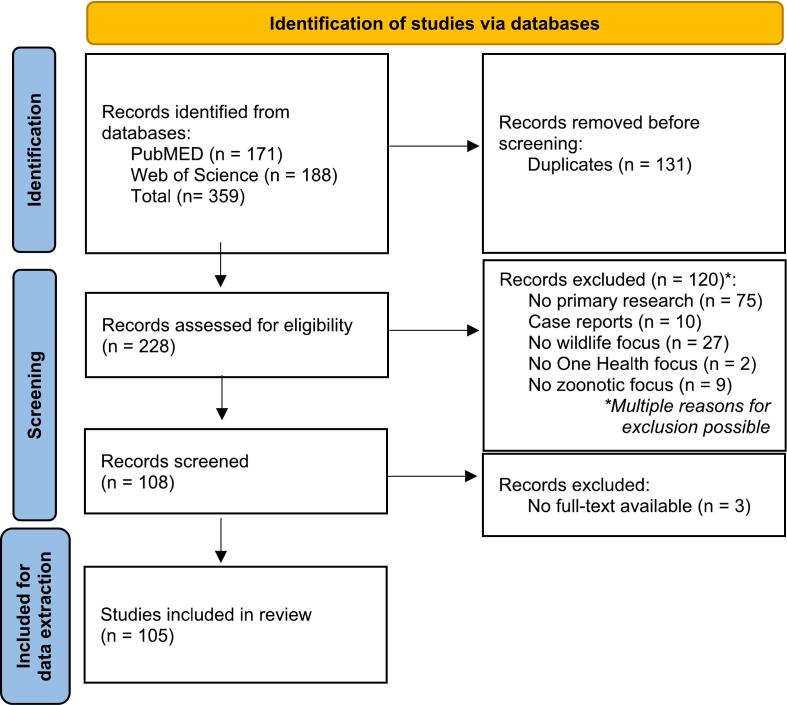
Preferred Reporting Items for Systematic Reviews and Meta-Analyses (PRISMA) flow diagram detailing number of records retrieved and selected for data extraction.

This systematic review was carried out with available literature online following the PRISMA guidelines [30]. A flow diagram of preferred reporting items is provided in Fig. 1.

### 2.1 Search strategy

The review was conducted by searching the two online databases PubMED and Web of Science for primary research papers on zoonotic diseases and wildlife. The review focused on the One Health approach and covered the period between January 2018 and September 2023. Database searches were performed on September 27th, 2023. The search terms used for the review are listed in Supplementary Table 1. Abstracts were retrieved and transferred into the Rayyan platform. Automatic deduplication was performed on the retrieved abstracts, and the remaining abstracts were manually validated.

### 2.2 Abstract screening for eligibility criteria

The titles and abstracts of each publication were screened by two reviewers independently. Inclusion was contingent upon the following criteria being met: The study must be primary research that focused on wildlife, addressed zoonotic diseases, and adopted the One Health approach. Only publications in English were included.

To be considered original research, studies had to involve the collection of primary research data. Consequently, studies that solely relied on secondary data or data obtained from publicly available databases or sources, such as veterinary offices or weather documentation, were excluded. Although case reports are typically classified as primary research, they were excluded because it is challenging to compare their study design to that of other research designs. In instances where information from the title and abstract was insufficient to distinguish primary data collection, the full text was consulted for a final decision on inclusion.

Publications were included if they provided data on wildlife or if the study aimed to generate findings impacting diseases in wildlife. Exclusions were made for resources where wild animals were only incidentally mentioned. Our definition of wild animals was broad, encompassing animals living in the wild or non-domesticated species in captivity, including feral animals and captive animals like farmed wildlife or zoo animals. Zoo animals were included in the review as they serve as models for their wild counterparts. Feral animals were considered due to their active role in wild zoonotic transmission pathways. Stray animals were excluded as they typically share habitats with humans and are socialized with them, unlike feral animals.

Publications with a focus on zoonotic diseases or those contributing to knowledge about zoonotic diseases through their methods and results were included. The key criterion for this focus was a thorough discussion of zoonotic dynamics rather than merely incidental reference. Furthermore, studies were included if they addressed a specific zoonotic pathogen or disease. Additionally, papers mentioning “One Health” in the title and/or abstract were included unless explicitly stated otherwise in the study itself. Papers with unavailable full text were excluded.

### 2.3 Data extraction

Following the initial screening, resources that met the inclusion criteria were selected for manual data extraction. Two researchers independently reviewed articles and recorded data in a Microsoft Excel sheet. In preparation for this task, we formulated and tailored definitions for (A) One Health domains and (B) study design. These definitions underwent a pilot test with ten articles. One Health domains, encompassing human, animal, and environmental health, were categorized according to the COHERE standards [24], further detailed by Cavalerie et al. [31]. The categorization occurred at two levels: (A1) Data Collection and (A2) Knowledge Generation. This distinction was based on the separation between sampling and knowledge generation within the same and other domains. The data collection for human health was further subdivided into social (e.g., conducting interviews) and biological sampling (e.g., blood samples). Accordingly, animal health was divided into biological and behavioral sampling, and environmental health into biotic and abiotic sampling. All animals, except vector-arthropods, were included in the animal domain. Because only studies that included wildlife were included in this review, the animals were either wildlife only animals or wildlife and domestic animals. Supplementary Table 2 provides detailed definitions for this categorization. On the level of knowledge generation, a resource was assigned to one or more One Health domains, if it contributed to or aimed to contribute to knowledge in this field.

An initial screening of the studies was performed to categorize the studies based on their design. The list provided by Cavalerie et al. [31] was adapted to the screened articles and for each study the following aspects were extracted: (B1) methods employed, (B2) quantitative or qualitative design, (B3) epidemiological design, and (B4) extent of non-academic participation and transdisciplinarity. A comprehensive approach to study categorization is presented in Supplementary Table 3.

(B1) The methods employed encompassed a diverse range of disciplines, including laboratory methods (molecular analysis, serological analysis, microscopy, in vivo testing, antimicrobial susceptibility testing, bacteriological or viral culturing), medical non-laboratory examination (clinical and pathological examination), social science methodologies (interviews, questionnaires, focus group discussions, and ethnography) and environmental studies (entomology, water/soil/effluents/food testing, ecological, and ethological methodologies). Although there may be overlaps and intersections between disciplines and methodologies, the variable *methods* serve as an indicator of interdisciplinarity.

(B2) Studies were categorized as quantitative if data handling was numbers-based, countable, or measurable, and qualitative if interpretation-based, descriptive, or language-related. Mixed-methods studies included both quantitative and qualitative data, indicating interdisciplinarity.

(B3) Epidemiological studies were classified as descriptive or analytical. Furthermore, their study desgin was divided into cross-sectional, longitudinal, case-control or other [32,33].

(B4) The involvement of non-academic stakeholders was categorized as an indication of the degree of transdisciplinarity and participation in the study, especially accounting for the degree of decision-making in the process [29].

### 2.4 Data analysis

The data extracted in Microsoft Excel 2016 were exported to R version 4.3.1 [34] for subsequent data cleaning and wrangling. This process resulted in suitable datasets for analysis. Descriptive tables were generated to summarize the distribution of variables across the One Health domains and study designs. Euler diagrams were employed with the *eulerr* package to visually present the distribution of data collection and knowledge generation across the Human, Animal, and Environmental domains. Following the conversion of the dataset to adjacency matrices, arc, and chord diagrams were constructed using the *circlize* and *ggplot2* packages to visually represent interdisciplinary collaborations and connections across different disciplines and methods utilized in the studies.

## 3 Results

### 3.1 One health domains

**Fig. 2:**
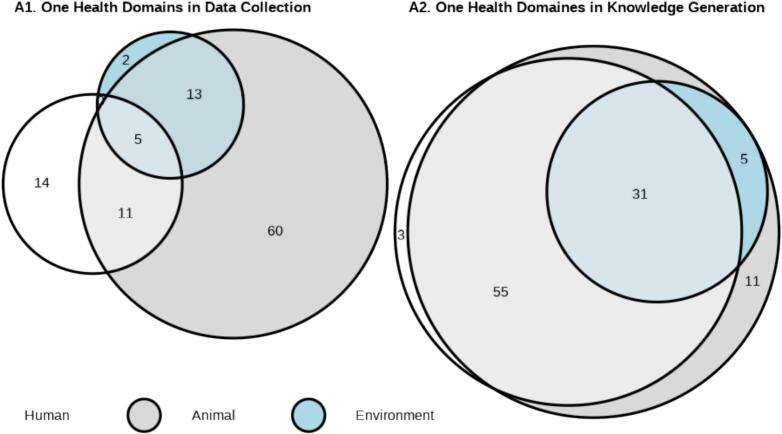
Overlap of Human, Animal and Environmental Domains in A1) Data Collection and A2) Knowledge Generation of the studies, visualized in Euler-diagrams.

A total of 228 primary research papers were retrieved. Out of these, 105 studies were included in the review. Five studies (4.8 %) obtained data from all three One Health domains - humans, animals, and the environment. Eleven studies (10.5 %) collected data from humans and animals, while 13 studies (12.4 %) collected data from animals and the environment (as shown in Fig. 2). In contrast, 31 out of 105 papers (29.5 %) generated knowledge for all three domains. In 60 papers (57.1 %), two domains were addressed - 55 papers studied animal and human health, while five papers studied animal and environmental health. The remaining 14 papers (13.3 %) generated knowledge for just one domain. Overall, the environmental domain was underrepresented in the studies included in this review with 20 (19 %) including the environment in data collection and 36 (34.3 %) creating knowledge for environmental health.

The most common form of data collection was the use of animal samples, which was presented in 89 papers (84.8 %). Of these, 87 studies collected biological samples, while eight studies focused on animal behavior. When examining the 30 publications with data collection within the human health domain in greater detail, it became evident that 13 publications focused on biological samples, while 20 studies included social parameters such as interviews. A total of 20 papers (19 %) included samples from the environmental domain, with eight focusing on biotic samples and 14 on abiotic samples. It is noteworthy that while knowledge generation was being addressed in 89 papers (84.8 %) on human health, 102 papers (97.1 %) on animal health, and 36 papers (34.3 %) on environmental health, data collection was less frequently addressed across all domains.

### 3.2 Study design

The studies analyzed employed primarily quantitative methods (*n* = 94; 89.5 %). Nine studies (8.6 %) employed both quantitative and qualitative methods, indicating a mixed-methodology approach. The remaining two studies (1.9 %) presented only qualitative data.

Laboratory methods were employed in the majority of studies (*n* = 87; 82.9 %), with molecular analysis (*n* = 72), serology (*n* = 34), microscopy (*n* = 21), in vivo testing (n = 3), antimicrobial testing (n = 21) and bacterial/viral culture (*n* = 20). It is noteworthy that the majority of studies employed more than one laboratory method. Medical examination were conducted by ten studies (9.5 %); nine involved animal sections taken for pathological examination, and one involved live clinical examination. Methods from the social sciences were represented in 20 papers (19 %). Interviews were conducted in six cases, questionnaires in 17 studies, focus group discussions in seven studies, and ethnography in three studies. Finally, entomology (*n* = 8), water/soil/effluent/food testing (*n* = 10), ecology (*n* = 9) and ethology (*n* = 11) were represented in the category of environmental methodology with a total of 29 papers (27.6 %).

**Fig. 3:**
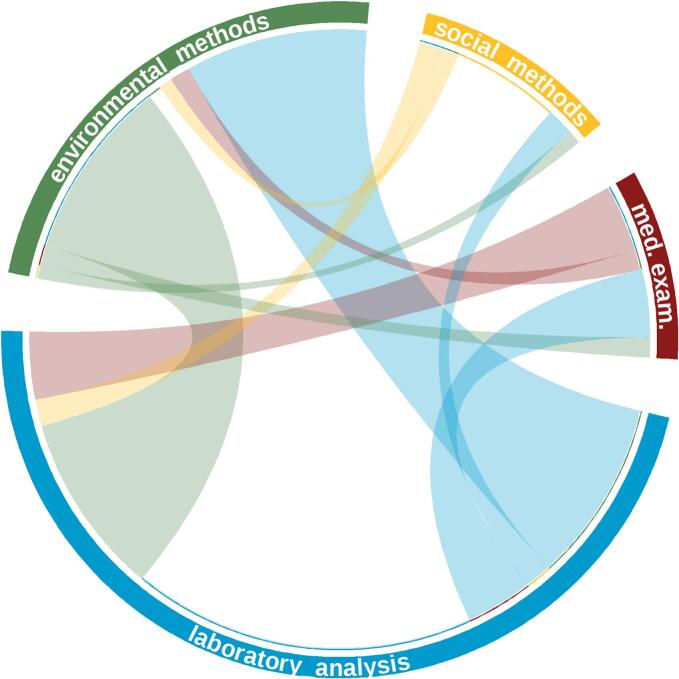
Chord diagram illustrating interconnections between methodologies employed across various disciplines in our study. The colors represent the disciplines itself, the thickness of the chords represents the frequency of connections, offering insights into methodological interdisciplinarity and convergence. Clear spaces within a discipline indicate self-links. Medical examination was abbreviated as *med. Exam*.

The chord diagram (Fig. 3) highlights the prioritized use of laboratory methods. Of the 87 papers (82.9 %) that used laboratory methods, 51 applied multiple laboratory methods. The strongest connections can be observed between laboratory and environmental methods, as evidenced by 27 papers. In ten papers, medical methods and in four papers, social sciences were combined with laboratory methods. All of the 10 papers that included medical examination in turn, overlapped with laboratory methods. Here, three additional connections to environmental methods can be reported. A sole use of environmental methods was reported in two studies, as these were mostly connected to laboratory methods. In contrast, the use of social science methods demonstrated a limited degree of connectivity, with just two instances of overlap with environmental sciences and four with laboratory methods.

**Fig. 4:**
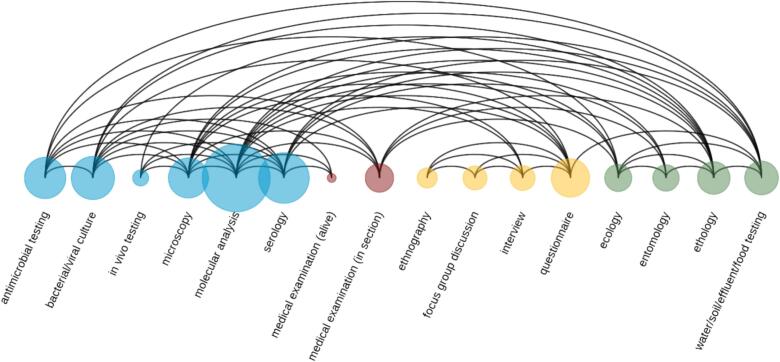
Network analysis of methodology applied in the studies: this arc diagram represents methods as nodes and their interconnections as arcs. The size of each node is proportional to the frequency of its application. Analogously to Fig. 3, the node colors represent the different disciplines: laboratory analysis (blue), medical examination (red), social methods (yellow), and environmental methods (green). (For interpretation of the references to colour in this figure legend, the reader is referred to the web version of this article.)

The arc diagram (Fig. 4) further details the interactions of applied methodologies, illustrating the network of connections between all subcategories and their interdisciplinary ties. It highlights the importance of molecular analysis in the studies included in this review and their combination with methods in other fields.

Of the 91 studies (86.7 %) that applied epidemiologic methods, 85 were cross-sectional studies, two were case-control studies, and four were longitudinal studies in design; 73 were descriptive and 18 were analytical.

Finally, 36.2 % of the publications included non-academic stakeholders (*n* = 38) and were thus classified as transdisciplinary. In four of these studies, stakeholders such as representatives of local communities, governmental or non-governmental institutions, and non-academic individuals who provided animals or data on animals (e.g., wildlife hunters) were actively involved in decision making or study design.

## 4 Discussion

Although the One Health approach is frequently mentioned in the studies included in this review, the findings indicate, that only a few studies combined human, animal, and environmental health. The high number of studies including animal sampling was not surprising given the topic of the review. Remarkably, especially in the field of data collection and reporting, only few studies include all of the three One Health domains.

No clear definition of One Health research as such was found, although reviews and analyses of research studies in the field referred to studies that mentioned the One Health approach [35], implying inter- and transdisciplinary collaboration, local and global, and encompassing the One Health domains of human, animal and environment [36]. Several authors pointed to a lack of criteria for One Health research studies [9,24]. Therefore, further clarification is needed in addition to existing frameworks, such as the COHERE standards, which underscore the importance of reporting data from all three One Health domains [24]. Cavalerie et al. [31], who found that only 4 % of studies on zoonoses between 1918 and 2018 reported on all three domains, expected an increase in the proportion of papers reporting data from humans, animals, and the environment simultaneously in the coming years. Based on our results, this expectation has not been met so far as only around 5 % of the papers met this criterion.

In knowledge generation, however, a greater overlap of domains was observed. These larger overlaps could be explained by the fact that some data sources contain information for more than their own domain. For instance, a molecular analysis of a blood sample from an animal may contain information about the pathogen spectrum and respectively the zoonotic potential for humans. On the other hand, it could also indicate that although all three domains are rarely sampled, a more complete One Health approach is aimed for, even if the initial data collection strategies may vary. The reasons for this may vary, but reducing the cost of sampling is certainly a factor.

Most studies generated knowledge on both animal and human health, but environmental health was still underrepresented. Although environmental change is discussed to be the most critical cause of zoonotic EID [1], this is not yet reflected in the collection of studies in this review. Similar observations have been made in previous reviews. Cavalerie et al. [31] found an underrepresentation of the environmental in studies of zoonotic diseases on the Horn of Africa, Schurer et al. [37] identified an isolation of the environmental sciences in studies on zoonotic parasites, and Schmiege et al. [38] found a focus on human-animal interactions in studies on COVID-19 and emphasized the importance of integrating environmental methods. It can therefore be assumed that the integration of environmental health should be further integrated into One Health research on zoonotic risks in wildlife. This is in line with *Action track 6* of the OH JPA, which emphasizes the need to integrate the environment into One Health [17]. The environment plays an essential role, particularly with regard to zoonotic diseases in wildlife. To move forward, One Health research needs to broaden perspectives beyond anthropocentric ontologies and strengthen collaboration with environmental sciences [39]. To strengthen wildlife and environmental health in One Health, it is suggested to recognize both socio-economic and environmental factors as foundations of health [40]. This process is expected to accelerate with the formal inclusion of UNEP in the Quadripartite (formerly Tripartite).

Social determinants of health have not only been identified as effective when addressed in Public Health interventions [41], but also as another neglected area within One Health research [42]. The results of this study can only partially support this finding, as social samples were more common than biological samples in the human domain of data collection. Overall, however, social science methods were applied less frequently than other disciplines, and one could therefore conclude that the social sciences are underrepresented and need to be better integrated to capture the complex socio-cultural factors that influence disease dynamics. Particularly noteworthy at this point is the sparse combination of social science methods with other disciplines and thus the low level of interdisciplinarity. In particular, the multi- or interdisciplinary integration of social sciences in the OH approach is crucial [37,43]. The social perspective could be used to identify risk behaviors for zoonotic transmission, and social science expertise can help in effective public communication and engagement. At the local level, social sciences such as anthropology are essential to understand cultural dynamics and ensure appropriate collaboration between communities and researchers [43,44]. In this review, only three studies employed ethnographic methods. In addition, most of the social science methods were quantitative. Overall, qualitative methods were rarely applied and only nine studies combined quantitative and qualitative methods. Although there is more to its interdisciplinary nature, mixed methods research is discussed as an integrative form of interdisciplinary research [26] and could therefore serve as an indicator of interdisciplinarity. Interdisciplinarity implies more than the selection of methods for a study. Of the three levels of interdisciplinary research design according to the MIR framework [26], we were only able to examine the technical design. The reason for this is that the way in which the researchers in the studies collaborated and their field of expertise is rarely mentioned in publications. Therefore, the nature and extent of interdisciplinarity was approached by categorizing methods and identifying mixed-method studies, but a more nuanced investigation may be necessary. This approximation is clearly one of the notable limitations of this review. Furthermore, the publication of truly interdisciplinary papers can be challenging, as it is difficult to find common journals Additionally, some disciplines, such as the social sciences, also produce book-like publications. In turn, the establishment of more interdisciplinary publication venues would support One Health research [45,46].

The results of this review especially highlight the interdisciplinarity of laboratory methods with the other disciplines, especially environmental and medical examinations, whereas the social sciences were more isolated in methodology. Most of the studies were classified as epidemiological studies, reflecting the field's utility in assessing zoonotic disease prevalence, spillover risk, and prevention measures. Cavalerie et al. [31] also found that most studies in zoonotic research in the Horn of Africa were epidemiological studies, with the majority being descriptive and observational, leaving room for analytical epidemiological studies in the field.

Several suggestions for overcoming silos have already been articulated and are applicable to the challenges identified in this review. A survey with researchers at the human-environment interface found that communication difficulties and lack of time, funding, and publication venues, were the most common barriers for interdisciplinary collaboration [45]. With regard to interdisciplinary communication, very different terminology and epistemological concepts need to be overcome, especially between natural and social scientists [47]. For a truly One Health research, it has been argued that One Health education and interdisciplinary training should be prioritized [46,48].

The review also identified a lack of non-academic stakeholder participation. Although one third of the publications included non-academic stakeholders, in only four of these studies the stakeholders from communities were actively involved in decision making or study design. To fully realize the transdisciplinary potential of One Health research, it is imperative to foster collaboration with diverse stakeholders, including non-academic entities and overcome the disconnection of institutions with society. This call for improvement is consistent with the proposition made by Wright et al. [29] in 2010. Today, in 2024, 14 years later, implementation is still lacking. The OH JPA emphasized the need for institutional support for capacity building and training of personnel across institutions that promote transdisciplinary collaboration and participatory research. Inclusive and equitable frameworks are needed that prioritize the equitable involvement of local communities in One Health initiatives [17]. We propose to follow this plan of action more closely, as it highlights the need for enabling policies and funding structures that promote the active participation of non-academic stakeholders. A participatory approach that incorporates community-driven insights and local knowledge is essential to bridge the gap between institutions and society, and to ensure that diverse voices shape One Health policies and address complex global health challenges.

## 5 Conclusion

This review highlights a persistent lack of integration of environmental health, social determinants of health, as well as methods from social and environmental sciences in the field of zoonotic diseases in One Health research. Despite the increased promotion of the One Health approach during the last years, this lack seems to persist, and it is essential to consider the implications of their underrepresentation. Neglecting these domains hampers our comprehensive understanding of disease transmission dynamics. Environmental factors, such as habitat destruction, climate change, and pollution, play a pivotal role in driving zoonotic disease emergence and spread. Failure to adequately account for these factors may lead to oversimplified models of disease transmission and hinder the development of effective prevention and intervention strategies. Similarly, social determinants of health, including socio-economic status, cultural practices, and access to healthcare, profoundly influence disease dynamics and community resilience. By neglecting these factors, One Health research runs the risk of overlooking key drivers of disease transmission and failing to address the root causes of health disparities. Furthermore, this review provides insight into the relationship between data collection and knowledge generation in primary One Health research studies. While there is a stronger aim to include all three One Health domains in knowledge generation, there is little integration of all domains in data collection. Despite the imperative for more interdisciplinarity in study design, the review also highlights the lack of transdisciplinarity and participatory approaches in One Health research on zoonotic diseases in wildlife, which limits the involvement of diverse stakeholders. Thus, inter- and transdisciplinarity and a holistic approach covering human, animal and environmental health in research on zoonotic diseases in wildlife is not only crucial for enhancing our understanding of disease dynamics but also essential for developing more effective, equitable, and sustainable public health interventions.

The following are the supplementary data related to this article.

Supplementary material 1 Final search terms (.docx). Supplementary material 1

Supplementary material 2 Definition of One Health Domains (.xlsx). Supplementary material 2

Supplementary material 3 Definitions of Study Design (.xlsx). Supplementary material 3

Supplementary material 4 List of papers (*n* = 105) included in the study with references (.xlsx). Supplementary material 4

## Funding

This systematic literature review is part of the study “Knowledge, attitudes, and practices towards the risk of zoonotic diseases, wildlife trade and wildlife consumption in Latin America” and financially supported by a fund of the German Federal Ministry for Economic Collaboration and Development (BMZ) through the International Alliance against Health Risks in Wildlife Trade and coordinated by the GIZ (Gesellschaft für 10.13039/100011259Internationale Zusammenarbeit).

## CRediT authorship contribution statement

**Caroline Kuhn:** Writing – original draft, Visualization, Software, Methodology, Investigation, Formal analysis, Data curation, Conceptualization. **Kenneth Mawuta Hayibor:** Writing – review & editing, Validation, Methodology, Investigation, Data curation, Conceptualization. **Ama Twumwaa Acheampong:** Writing – review & editing, Validation, Investigation. **Luciana Salini Abrahão Pires:** Writing – review & editing, Validation. **Magda Clara Vieira Costa-Ribeiro:** Writing – review & editing, Validation. **María Soledad Burrone:** Writing – review & editing, Validation. **Carlos Roberto Vásquez-Almazán:** Writing – review & editing, Validation. **Katja Radon:** Writing – review & editing, Validation, Supervision, Resources, Methodology, Conceptualization. **María Teresa Solis Soto:** Writing – review & editing, Project administration, Funding acquisition. **Abrahão Pires Luciana Salini:** Writing – review & editing, Validation. **Adler Marcia:** Writing – review & editing, Validation, Investigation. **Burrone María Soledad:** Writing – review & editing, Validation. **da Costa Ribeiro Magda Clara Vieira:** Writing – review & editing, Validation. **de Almeida Gustavo Araújo:** Writing – review & editing, Validation, Investigation. **de Carvalho Denise Siqueira:** Writing – review & editing, Validation, Investigation. **de Tarso Pires Paulo:** Writing – review & editing, Validation, Investigation. **Encina Zamorra Veronica:** Writing – review & editing, Validation, Investigation. **Garrido Marie Astrid:** Writing – review & editing, Validation, Investigation. **Guzmán-Quilo Maria Carolina:** Writing – review & editing, Validation, Investigation. **Kuhn Caroline:** Writing – original draft, Visualization, Software, Methodology, Investigation, Formal analysis, Data curation, Conceptualization. **Magalhães Buffon Marilene da Cruz:** Writing – review & editing, Validation, Investigation. **Mansilla Vivar Pilar Macarena:** Writing – review & editing, Validation, Investigation. **Mendez Heredia Dennis Martin:** Writing – review & editing, Validation, Investigation. **Perez Morales Fabiana Marcela:** Writing – review & editing, Validation, Investigation. **Pinto Navia Carlos Fernando:** Writing – review & editing, Validation, Investigation. **Radon Katja:** Writing – review & editing, Validation, Supervision, Resources, Methodology, Conceptualization. **Ribeiro de Almeida Tatjana Queiroz:** Writing – review & editing, Validation, Investigation. **Solis Soto María Teresa:** Writing – review & editing, Project administration, Funding acquisition. **Vásquez-Almazán Carlos Roberto:** Writing – review & editing, Validation.

## Declaration of generative AI and AI-assisted technologies in the writing process

During the preparation of this work the authors used DeepL Write and ChatGPT, Version 3.5–4 in order to enhance the clarity and coherence of the text, ensuring effective communication of ideas and concepts. After using this tool, the authors reviewed and edited the content as needed and take full responsibility for the content of the publication.

## Declaration of competing interest

The authors declare that they have no known competing financial interests or personal relationships that could have appeared to influence the work reported in this paper.

## Data Availability

I have shared data in the supplementary files.
