# Supplementary material for: How studies on zoonotic risks in wildlife implement the one health approach – A systematic review
Source: One Health. 2024 Nov 8;19:100929. doi: 10.1016/j.onehlt.2024.100929 (PMC11582546; doi:10.1016/j.onehlt.2024.100929)
Supplement: Supplementary material 1 — Final search terms (.docx). [file mmc1.docx]

# Search Terms

PubMED search on 2023-09-27 (171 results):

((("zoono*"[Title/Abstract] AND "wild*"[Title/Abstract] AND "one health *"[Title/Abstract]) NOT ((("Review"[Publication Type] OR "systematic review"[Publication Type] OR "Address"[Publication Type] OR "Autobiography"[Publication Type] OR "Bibliography"[Publication Type] OR "case reports"[Publication Type] OR "Comment"[Publication Type] OR "Directory"[Publication Type] OR "Editorial"[Publication Type] OR "Festschrift"[Publication Type] OR "government publication"[Publication Type] OR "Guideline"[Publication Type] OR "historical article"[Publication Type] OR "Interview"[Publication Type] OR "Portrait"[Publication Type] OR "personal narrative"[Publication Type] OR "News"[Publication Type] OR "newspaper article"[Publication Type] OR "Legislation"[Publication Type]) NOT "legal case"[Publication Type]) OR "Letter"[Publication Type])) AND 2018/01/01:2023/12/31[Date - Publication]) NOT "Review"[Title/Abstract]

Web of Science search on 2023-09-27 (188 results):

(TI=(zoono*) OR AB=( zoono*)) AND (TI=( wild*) OR AB=( wild*)) AND (TI=("One Health") OR AB=("One Health")) and Article (Document Types) NOT TI=(Review)) NOT AB=(Review)
